# Development and Delphi validation of instrument for the preparation of a GMP audit of a cosmetic contract manufacturer in the UAE

**DOI:** 10.1038/s41598-022-14457-7

**Published:** 2022-07-04

**Authors:** Ammar Abdulrahman Jairoun, Sabaa Saleh Al-Hemyari, Moyad Shahwan, Faris El-Dahiyat, Justyna Bisgwa, Shazia Jamshed, Hanady Yousef Shourrab

**Affiliations:** 1 Health and Safety Department, Dubai Municipality, Dubai, United Arab Emirates; 2 School of Pharmaceutical Sciences, Universiti Sains Malaysia, Penang, Malaysia; 3 Pharmacy Department, Emirates Health Services, Dubai, United Arab Emirates; 4 Department of Clinical Sciences, College of Pharmacy and Health Sciences, Ajman University, Ajman, United Arab Emirates; 5 Centre of Medical and Bioallied Health Sciences Research, Ajman University, Ajman, United Arab Emirates; 6 Clinical Pharmacy Program, College of Pharmacy, Al Ain University, Al Ain, United Arab Emirates; 7 AAU Health and Biomedical Research Center, Al Ain University, Abu Dhabi, United Arab Emirates; 8 Opole, Poland; 9 Department of Clinical Pharmacy and Practice, Faculty of Pharmacy, Universiti Sultan Zainal Abidin, 21300 Kuala Terengganu, Malaysia; 10 Drug Department, Ministry of Health and Prevention, Dubai, United Arab Emirates

**Keywords:** Environmental sciences, Environmental social sciences

## Abstract

Outsourcing is rapidly increasing in the cosmetic industry. When cosmetic manufacturing activities are outsourced, product quality control and assurance procedures are important to guarantee the safety of the final products. One method to ensure the compliance of potential contract manufacturers with Good Manufacturing Practices (GMP) is auditing. Auditing can be confirmed by using GMP questionnaires. The current study aims to develop an effective instrument, in the form of a questionnaire, and to validate its content using the Delphi method. A modified Delphi method used an expert panel to develop a questionnaire-based audit preparation instrument. In the Delphi questionnaire round, 50 experts from cosmeceutical industries, relevant authorities, and universities assessed the content validity of the audit preparation instrument. The Delphi questionnaire round targeted 70 experts from the cosmetics industry; 50 completed the questionnaire, giving a response rate of 71.4%. Their agreement level on the quality of the instrument items ranged between 56 and 96%. Of the 52 items, 47 (90.4%) met the predefined criterion for an agreement rate of at least 75%. The proposed audit preparation instrument demonstrated good content validity, and the expert panel participating in the Delphi questionnaire round made a few minor suggestions for modifications. The modified Delphi method used for the content validation of the instrument proved to be suitable. However, based on the panel’s feedback, additional research is needed to ensure the maximum applicability and practicality of the instrument.

## Introduction

Cosmetics and personal care products are among the most popular consumer goods and are being sold in increasing amounts, with an average annual increase in the global cosmetics market of around 5%. The growth of this market has remained relatively stable over the years, even remaining strong under unstable economic conditions^1^**.** Due to the increasingly young population, the demand for cosmetics in the United Arab Emirates (UAE) market is predicted to reach $3 billion by 2025. With its strong gross domestic product (GDP) and high living standards, Dubai’s population was the largest consumer of cosmetics in the UAE until 2019. Abu Dhabi, the second-largest market in the UAE, similarly experienced a high demand for such products due to its growing population and GDP as well as the entry of international brands to the market. In 2020, the COVID-19 pandemic led to explosive growth in online shopping as consumers avoided physical stores due to safety concerns and restrictions, and the purchase of cosmetics products was no exception^2^**.**

Trends in cosmetics consumption are unlike those of pharmaceuticals, which are normally only bought by those who are sick or in need of some form of treatment. By contrast, cosmetics and personal care products are important in everyday life. Cosmetics manufacturers are building on their growing popularity, aiming to increase their profits; however, this can lead to the sale of unsafe products that have negative side effects for the consumer. To ensure the safety of the final product, quality control is particularly important for cosmetics and personal care products. Current cosmetics regulations in the UAE state that GMP certification is obligatory for manufacturers of cosmetics, and the Dubai Municipality & Emirates Authority for Standardization & Metrology (ESMA) requires a GMP certificate for product registration^3,4^**.** These measures are intended both to stop unsafe products from entering the market and to instruct users on the safe use of approved products. Thus, each cosmetic product on the Dubai market must be registered and adhere to government regulations concerning its manufacture, sale, and import (if applicable). Furthermore, businesses handling cosmetic products must be licensed, and local cosmetics manufacturers must have ISO 22716 certification to ensure that the products have been produced using GMP. This certification outlines the requirements for manufacturers, including production facilities, quality and safety, equipment and packaging, production processes, laboratory controls, and staff management. GMP is a system based on available quality management systems, procedures and instructions that are to ensure the correct quality of the final product and safety in its use. Personnel should have knowledge of the production, control and storage of products of a certain quality. All premises must be properly designed and operated in order to maintain them in an appropriate cleanliness, as well as to facilitate the location and protection of raw materials and finished products. Equipment should be used for its intended and specific purpose. It is important that equipment is not difficult to clean and disinfect. In the case of using automated systems, appropriate supervision over their correct functioning should be introduced. Raw materials and packaging materials must meet all acceptability criteria, appropriate for the quality of individual finished products, and the actions taken at each stage of the product must take into account the characteristics of the finished product and follow the given procedures. The finished product must meet all quality criteria, and the method of its storage, return and transport must not affect its acceptability. In the quality control laboratory, all policies relating to personnel, equipment, rooms and documentation should be followed. The employees of this department are also obliged to perform all checks and analysis of samples in order to determine their acceptability. All complaints and withdrawals from the market should be subjected to research and appropriate analysis, on the basis of which decisions on further proceedings will be made and, when necessary, corrective actions should be taken. Each organization is required to create its own documentation system, which should contain information on all activities related to the implementation and maintenance of the system. The benefits that the manufacturer can bring from the implementation of GMP are: improvement of the quality of finished products and the functioning of the organization; clear and comprehensive documentation system; reducing the risk of placing a dangerous unsafe product on the market; increasing the competitiveness of the entity and its brand(s); increase in customer confidence; increasing employee awareness; extending the life of devices through their proper operation and maintenance; fast detection of non-compliance and reacting to the possibility of their occurrence by introducing controls (audits); guarantee of manufacturing products that will be safe for human health when used under normal or reasonably foreseeable conditions of use^5^**.**

The major challenge facing the cosmetics sector is manufacturing high-quality and safe cosmetics while also tackling rising production costs. To survive, manufacturers are relying on their existing core competencies and developing ways to overcome their non-core competencies. In effect, this means that many are turning to outsourcing. While ad hoc outsourcing remains crucial, the trend in the cosmetics industry is towards strategic outsourcing. However, several UAE cosmetics companies lack a defined protocol for selecting and managing contract manufacturers, even though there is a clear need to control outsourced production, especially as the marketing authorization holder retains the final responsibility for product quality^6^**.** Hence, the best possible contractor must be chosen, and substandard efforts in making this choice in the past have led to significant cosmetic product recalls.

A recent analysis of 102 alcohol-based hand sanitizers on the market in Dubai found that 6 contained undeclared methanol, while others revealed alcohol content significantly below 60%, despite being labelled as 70% alcohol^7–9^**.** Similarly, in Ajman, also in the UAE, two factories were closed after substantial amounts of fake medical sterilizers were found on the premises. The product labels had been removed, and the actual content was a perfumed body spray^10^**.** Numerous such cases of adulterated or contaminated cosmetic products have occurred in the UAE. For example, research analysing 100 cosmetic and personal care products on the UAE market found that 13% had been contaminated by yeast or mould, while 5% contained aerobic mesophilic bacteria^11^**.** Similar research in the same context found that 13% (n = 9) of sampled products not only contained dangerous levels of formaldehyde but also did not declare free formaldehyde or formaldehyde releasers on the content labels^12^**.**

When manufacturers decide to outsource, they must first evaluate potential contractors. In particular, contractors must be audited in terms of their GMP^6,13,14^**.** Furthermore, even if the contractor is audited prior to the start of production, their activities must be continuously evaluated during the production process^6,13–15^**.** However, before the outsourcing company can initiate the auditing, initial research must be conducted, such as via questionnaires^14^**.** To ensure a high-quality audit, a substantial amount of background information must be obtained and integrated into the planning of the audit agenda or checklist^16^**.** In light of this, the current study aims to develop an effective instrument, in the form of a questionnaire that can assist in the preparation of the cosmetic contract manufacturer auditing process. This questionnaire primarily contains questions relevant to ISO 22716 GMP and is aimed at gathering preliminary information on potential cosmetic contract manufacturers to better prepare for the audit process.

## Methods

### Study design

The current study aimed to develop and validate an effective instrument that can assist in the preparation of the cosmetic contract manufacturer auditing process in the UAE. This study employed a modified Delphi method. This method enables group judgment on a subject that lacks accurate and/or complete information drawn from research^17,18^**.** A Delphi survey involves a set of expert panelists completing and evaluating a questionnaire in rounds, thereby successively highlighting those items for which there is high agreement, i.e., consensus. After the responses have been summarized after each round, those items on which the panelists were unable to agree are removed from all subsequent questionnaire rounds. This process enables the continuous refinement of the consensus among experts on the given topic. As participant anonymity is ensured throughout the procedure, this method enables controlled feedback to be obtained^19^**.** This method is often used to develop consensus on guidelines or standards for which there is little or no research-based evidence^20^**.**

### Recruitment of participants

The following two participant types were included in this study:

#### Scientific committee

Initially, a scientific committee was set up. This consisted of seven experts, with three of these coming from the relevant regulatory authorities while the remaining four were specialists attached to Ajman and Al Ain universities. To be chosen for the scientific committee, the members needed to have a PhD as well as a strong record of experience and knowledge in the following fields: the cosmetics industry, pharmaceutical technology and good manufacturing practice auditing (GMP). Furthermore, we included 10 experts from the case company, a medium-sized cosmetics producer that uses several contract manufacturers for its finished products. The aim hereby was to firstly, ensure that the scientific committee had sufficient expertise and experience on the investigated topic and secondly, prevent the findings from being unrepresentative of the larger population.

Next, the scientific committee conducted a review of the questionnaire’s first version developed by the researchers. The members were hereby asked to recommend changes to the questionnaire before it was sent to the panel of experts. They were also requested to evaluate the criteria that were used to choose which experts should take part in the Delphi rounds. They could also recommend additional members, as long as these fulfilled the selection criteria. The professionals suggested by the research team were then sent invitations asking them to join the scientific committee. Upon agreeing to take part, each committee member received the relevant documents by email for them to review.

#### Expert panel

The selection of experts can be done based on very few guidelines or rules^21^**.** Generally, an expert group can be assembled from individuals who have relevant knowledge or expertise, e.g. academics or specialists, or professionals who are active in the field^22^.

To be chosen for the Delphi panel, potential respondents had to be experts, either international or national, and possess the right skills and knowledge in the auditing of good manufacturing practices in the cosmetics and personal care product sector, demonstrated by a proven record of scientific papers published in peer-reviewed academic journals or policy reports; participation at cosmeceutical science conferences was also considered as relevant. Furthermore, the respondents needed to be proficient English speakers and able to complete the questionnaire in English without the need for translation. In particular, each panel expert needed to be able to do perform the following:

- Clarify the principles, processes and techniques of GMP and their importance for the cosmetics and personal care products industry.
- Perform third-party audits in the cosmetics and personal care products industry as per ISO 22716 GMP.

### Sampling and sample size

As there are few guidelines indicating how many experts are ideally required to be on a Delphi panel, the researchers settled on at least 20 experts^23,24^. Consequently, 70 experts were contacted and asked if they would agree to participate in the Delphi consensus of whom 50 expressed interest. Various professionals with deep knowledge of ISO 22716 GMP auditing and certification were chosen for the expert panel using purposive sampling. They were then registered on the researchers’ network and sent an email inviting them to take part. If they agreed, the link to the online questionnaire was sent to the respondents via email. The cover page of the questionnaire provided information on the study’s purpose as well as the risks and benefits to the participants. By completing the questionnaire, the participants were assumed to have given their informed consent.

### Development of the audit preparation instrument

To begin with, the extant literature on the topic was reviewed by first performing a search of full-text publications on the Scopus and PubMed databases. The topic was evidence of best practices in ISO 22716 GMP auditing. Once studies had been identified, relevant information was extracted on the best practices for optimal cosmetic products production, control, storage and shipment. An Excel spreadsheet was used to collate all relevant statements. The items for the first Delphi questionnaire version were developed based on the literature review results. These were then sent to the scientific committee for their opinion, after which a brainstorming session was held to address any issues. The final draft consensus document comprised an instrument for evaluating the contract manufacturers of sterile as well as non-sterile finished cosmetics products.

### Delphi questionnaire round

This study employed a one modified round Delphi survey; this is considered suitable if there is a considerable body of primary literature on the topic being investigated^25^**.** The draft version of the questionnaire comprised a list of statements sent via email to the 50 panel members. In the document, the study objectives were clearly described, and specific instructions on participation were included. The draft of the audit preparation instrument was sent to the panel experts in February 2021, and they were asked to evaluate it by ranking each item’s significance on a 5-point Likert scale (from 1 = strongly disagree to 5 = strongly agree). The panel experts also gave their opinions on whether any items were missing from the instrument and whether they could suggest any further modifications or had any other feedback on the questionnaire’s content. The respondents could clarify their responses using the text fields located at the end of each section. The Delphi round aimed to also highlight any redundancies or identify issues that influenced the comprehension of each individual statement. A research assistant calculated each item’s response frequencies and anonymously recorded them in a database. The following consensus criterion was used: if more than 75% of the panel ranked an item as either (agree) or (strongly agree), it was considered essential and retained in the instrument. If this criterion was not met, the item was considered non-essential and excluded. There have been similar expert consensus definitions used throughout the literature^26–28^.

### Data collection

The Google Online Survey tool was employed to operate the online questionnaire’s Delphi round. An email, containing an introductory letter as well as the link to the online questionnaire, was sent to the chosen experts. The experts were first asked to answer a question on whether or not they were willing to participate in the questionnaire, and only those who indicated their willingness were asked to fill in the questionnaire.

Two reminders, sent two weeks apart, were sent to any non-responsive experts. The data were collected between February 2021 and April 2021.

### Statistical analysis

The data were analysed using SPSS version 26. The sample’s demographics and baseline characteristics were summarized using frequencies and percentages. Mean ± Standard Deviation (± SD) was used to summarize the quantitative variables (GMP items). Differences in agreement level between groups were evaluated using Mann–Whitney and Kruskal–Wallis tests. A p-value of less than 0.05 was selected to demonstrate statistical significance.

### Ethics approval and consent to participate

This study received approval from Ajman University’s Institutional Ethical Review Committee (P-H-S-2021-2-9). All methods were carried out in accordance with relevant guidelines and regulations. The study aim was clearly presented on the questionnaire cover page, and all respondents were informed that their participation was completely voluntary. If the Participants proceeded to the second page of the questionnaire, were considered to have given their written informed consent. The participants’ identities were not recorded and the confidentiality of their data was guaranteed.

## Results

### Baseline characteristics of the participants

Table 1 shows the demographic and baseline characteristics of the different types of experts who participated in the study. A total of 50 professional experts participated in the panel. Of these, 10% (n = 5) had less than 1 year of work experience, 50% (n = 25) had 1 to 5 years work experience, 16% (n = 8) had 6–10 years work experience, 8% (n = 4) had 11–15 years work experience and 16% (n = 8) had ≥ 16 years. Among the participants, 20 (40%) had bachelor’s or equivalent degrees, 27 (54%) had master’s degrees and 3 (6%) held PhDs. Slightly more than half (54%) worked in public (government) organization, 36% in private organizations and 10% worked in both public and private organizations. The roles of the experts were as follows: 6 (12%) R&D Product Specialists, 6 (12%) Quality Control Supervisors, 10 (20%) Quality Assurance Specialists, 13 (26%) Production Technologist and ISO Representatives, 4 (8%) ISO 22,716 Auditors, 3 (6%) Data Entry Encoders, and 8 (16%) Cosmetic Safety Assessors. Of the participants, 70% (n = 35) were from the UAE and 30% (n = 15) from the EU.

**Table 1:** Expert panel participants’ baseline characteristics (n = 50).

Baseline	Groups	Frequency	Percentage
Work experience			
	Less than 1 year	5	10%
	1–5 years	25	50%
	6–10 years	8	16%
	11–15 years	4	8%
	16 years or above	8	16%
Education level	Bachelor’s or equivalent	20	40%
	Master’s or equivalent	27	54%
	PhD	3	6%
Organization type	Public (government)	27	54%
	Private	18	36%
	Both	5	10%
Position	R&D specialist—product	6	12%
	Quality control supervisor	6	12%
	Quality assurance specialist	10	20%
	Production technologist & ISO representative	13	26%
	ISO 22716 auditor	4	8%
	Data entry encoder	3	6%
	Cosmetic safety assessor	8	16%
Region (country)	UAE	35	70%
	EU	15	30%

*R&D* Research and Development, *UAE* United Arab Emirates, *EU* European Union.

### Phase 1: developing the audit preparation instrument

To pinpoint evidence of best practices in ISO 22716 GMP auditing, a literature search was conducted, whereby 22 relevant publications were found. Following a title and abstract screening, 14 of the 22 were chosen and the scientific committee submitted a further 4 . As a result, the study was able to identify 18 publications on the production, control, storage and shipment of cosmetic products.

The Guidelines on Good Manufacturing Practices (ISO 22716: 2007) were initially used to create the framework. These were specifically formulated to ensure that the cosmetics industry implements good manufacturing practices. Hence, they outline cosmetic products’ pharmaceutical supply chains and are applicable to all supply chain members, from the raw material producers to the manufacturers of cosmetics products. Specifically, they explicate the special requirements to be considered in constructing a quality management system (QMS) in accordance with the principles of GMP and ISO 22716.

**Figure 1:**
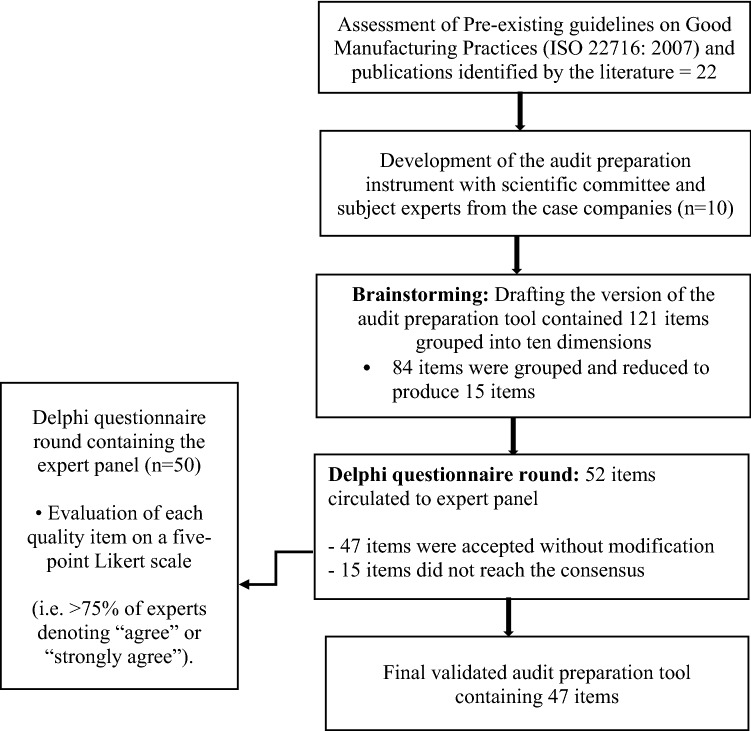
Audit preparation tool development.

Aligning with the ISO 22716: 2007 taxonomy, the brainstorming session with the scientific committee determined ten dimensions for GMP in the cosmetics manufacturing industry (Table 2). These encompassed production, raw materials, premises, equipment, personnel, laboratory control, labelling and packaging, customer complaints, recording, and others. Subsequently, the scientific committee convened to come to an agreement on these categories and describe each one according to their expertise. The brainstorming session used the draft audit preparation instrument, which mainly measured the items using closed-ended questions, i.e. the responses were in the yes/no format. This was chosen after consultation with professionals from the case company, and it has been established that this method is convenient for respondents to give their opinions. Using several open-ended questions in the questionnaire could have burdened the respondents due to the time needed to complete it. Following the brainstorming session an effort was made to identify and merge or remove redundant statements as well as statements that incorporated similar constructs. Hereby, of the initial 121 statements, 84 were either removed or grouped, eventually producing 15 statements with consensus for incorporation into the draft audit preparation instrument. Overall, 52 statements were taken to be used in the draft audit preparation instrument. Building on the Assessment of pre-existing guidelines on Good Manufacturing Practices (ISO 22716: 2007) as well as the articles gathered during the literature search, the final draft audit preparation instrument comprised 52 items categorized in the abovementioned ten dimensions. During the Delphi round voting process, these 52 statements were sent to the experts on the panel (Fig. 1).

**Table 2:** Experts’ agreement rating of the GMP: ISO 22716: questionnaire items (n = 50).

GMP items	Agreement rate, n (%)
**Premises:** **building** **and** **facilities:** **check** **whether**	
1. Premises are maintained in good repair and suitable for cosmetic production	38 (76%)
2. There is adequate lighting and ventilation	39 (80%)
3. Cosmetic materials, utensils, cosmetic contact surfaces of equipment, or finished products are not able to be contaminated by any condensate	36 (72%)^a^
4. Water supply; drainage enables sanitary operation both of equipment and personal cleanliness	44 (88%)
**Equipment:** **check** **whether**	
1. Equipment and utensils used in manufacture are of appropriate design and construction to prevent corrosion, build-up of material and cross-contamination from lubricants, dirt and sanitising agents	45 (90%)
2. Equipment in direct contact with product must be cleaned, maintained and sanitized at regular intervals	46 (92%)
3. Cleaned and sanitized equipment is suitably stored to protect from splash, dust and other contamination	45 (90%)
**Personnel:** **check** **whether**	
1. All personnel supervising and/or manufacturing product have the appropriate knowledge and training	47 (92%)
2. All persons coming into contact with cosmetic materials and finished product wear suitable protective clothing and maintain personal cleanliness	44 (88%)
3. Food and drink and use of tobacco are restricted to designated areas	47 (94%)
**Raw** **materials:** **check** **whether**	
1. Raw materials and packaging are stored and handled correctly to prevent cross contamination and decomposition from extreme conditions	48 (96%)
2. Raw materials are kept in sealable containers and stored off the floor	45 (92%)
3. Raw materials are labelled to clearly show identity, lot/batch identification and control status	49 (96%)
4. Raw materials are regularly sampled and tested to ensure the absence of contamination with microorganisms and other substances to prevent adulteration of finished product. Particular attention to be paid to vegetable oils and materials obtained by the cold process method	44 (88%)
5. Materials not meeting accepted guidelines are clearly identified and disposed of to prevent further use in cosmetic products	40 (80%)
**Production:** **check** **whether** **manufacturing** **and** **control** **have** **been** **established** **and** **written** **instruction**	
1. Equipment for processing, transfer and filling, and containers for raw and bulk materials are clean and in good repair	46 (94%)
2. Only approved materials are used	45 (90%)
3. Samples are taken during and/or after manufacture for testing for adequacy of mixing, absence of hazardous microorganisms and chemical contaminants in accordance with accepted specification	47 (94%)
4. Weighing and measuring of raw materials is checked by a second person, where possible, and all containers holding materials are clearly marked	40 (82%)
5. Equipment, containers and tanks used for processing, filling and holding cosmetics are identified to indicate contents, batch, and all relevant information	48 (94%)
6. Labels are identified correctly before labelling to avoid mix-up	46 (92%)
7. Equipment for processing, holding, transferring and filling of a batch is labelled regarding identity, batch and control status	47 (94%)
8. Packaging of finished products are clearly identified with permanent code marks	44 (88%)
9. Returned cosmetic products are examined for deterioration and contamination	41 (82%)
**Laboratory** **controls:** **check** **whether**	
1. Raw materials, in-process samples and finished products are tested to verify their identity and to determine compliance with specifications	45 (92%)
2. Reserve samples of lots/batches of raw materials and finished product for a specified time period, stored under conditions to protect from contamination or deterioration, and retest for continued compliance within accepted specifications	45 (90%)
3. Water supply is tested regularly for conformance with chemical-analytical and microbiological specifications	46 (90%)
4. Fresh and retained samples of finished products are tested for adequacy of preservation against microbial contamination within foreseeable conditions of storage and consumer use	44 (88%)
**Records:** **check** **whether** **control** **records** **are** **maintained** **of**	
1. Raw materials and primary packaging materials, documenting disposal of rejected materials	41 (82%)
2. Every batch made, with corresponding batch code, date of manufacture and quantity made	45 (90%)
3. Stock control of both raw materials and finished products	42 (86%)
4. Records are kept for the appropriate length of time, as specified by EU cosmetics regulation 1223/2009	43 (86%)
5. Manufacturing of batches, documenting Kinds, lots and quantities of material used	46 (92%)
6. Manufacturing of batches, documenting Processing, handling, transferring, holding and filling	47 (94%)
7. Manufacturing of batches, documenting Sampling, controlling, adjusting and reworking	44 (90%)
8. Manufacturing of batches Code marks of batches and finished products	48 (94%)
9. Distribution, documenting initial shipment, code marks and consignees	44 (88%)
**Packaging** **and** **labelling:** **check** **whether**	
1. Product containers are appropriate for the product contained	44 (88%)
2. Name of product and the net contents	43 (86%)
3. Name and address of the manufacturer of the product	44 (88%)
4. The INCI list of ingredients	40 (80%)
5. Allergens declaration as required by EU cosmetics regulation 1223/2009	39 (78%)
6. Any warning statement necessary or appropriate to prevent any pre-determined health hazard	43 (86%)
7. Direction for safe use of product	44 (88%)
8. For hair dye products, a cautionary statement and appropriate directions for preliminary patch testing	38 (76%)
**Complaints:** **check** **whether** **the** **firm** **maintains** **a** **consumer** **complaint** **file** **and** **determine**	
1. The kind and severity of each injury and body part involved	38 (76%)
2. The product associated with each injury, including batch code number	42 (84%)
3. Medical treatment involved, if any, including the name of attending physician	28 (56%)^a^
4. Name(s) and location(s) of any poison control center, government agency, physician’s group etc., to whom information and/or toxicity data are provided	29 (58%)^a^
**Other:** **check** **whether** **the** **firm** **is**	
1. Participating in the program of registration via the Cosmetic Products Notification Portal (CPNP)	34 (68%)^a^
2. Not using a colour additive not listed or certified for use in cosmetics. This includes colour additives used by suppliers	35 (72%)^a^
3. Not using a prohibited cosmetic ingredient, either by the firm or the supplier	40 (80%)

^a^Agreement rate below the pre-defined criteria and item excluded from the final audit preparation tool.

### Phase 2: Delphi questionnaire round

Following the voting for Delphi round, the experts agreed upon 47 of the 52 statements (i.e. > 75% of experts denoting “agree” or “strongly agree”). The final guideline document thus incorporated these 47 statements. The panel also reached consensus to omit five items from the final document. The mean score distributions for the individual dimensions of the questionnaire are given in Table 3.

**Table 3:** Descriptive statistics for the questionnaire dimensions.

Construct	No. of items	Mean	± SD
Premises: building and Facilities	4 items	79	30.03
Equipment	3 items	90.6	22.37
Personnel	3 items	91.33	22.14
Raw materials	5 items	90.40	19.05
Production	9 items	90	18.13
Laboratory controls	4 items	90	23.14
Records	9 items	89.11	19.24
Packaging and labelling	8 items	83.75	29.47
Complaints	4 items	68.5	36.70
Other	3 items	73.3	35.63
Overall	52 items	85.57	17.35

Table 4 presents the distribution of the agreement rate in association with the demographic details and baseline characteristics of the respondents. There were no statistically significant differences in the agreement rates originating from the demographic information or baseline characteristics of the respondents.

**Table 4:** Overall agreement rate stratified by experts’ baseline characteristics.

Baseline	Groups	Mean	± SD	Median	p-value
Work experience	Less than 1 year	36.20	21.04	43	0.145
	1–5 years	43.96	7.20	46	
	6–10 years	45.37	5.97	47	
	11–15 years	48.25	6.18	51	
	16 years or above	48.63	2.87	49.5	
Education level	Bachelor’s or equivalent	43.40	11.64	46.5	0.676
	Master’s or equivalent	44.93	7.09	47	
	PhD	48.0	4.35	50	
Organization type Position	Public (government)	45.77	10.16	48	0.551
	Private	42.77	7.86	46	
	Both	43.80	6.22	40	
	R&D specialist—product	41.83	4.75	39.5	0.684
	Quality control supervisor	49.50	2.59	50	
	Quality assurance specialist	44.90	5.74	46.5	
	Production technologist & ISO representative	45.07	13.85	49	
	ISO 22716 auditor	39.25	8.65	36	
	Data entry encoder	41.66	9.24	47	
	Cosmetic safety assessor	45.0	8.55	47.5	
Region (country)	UAE	45.60	9.74	49	0.191
	EU	41.93	6.68	43	

*R&D* Research and Development, *UAE* United Arab Emirates, *EU* European Union.

## Discussion

The establishment of the ISO 22716 GMP guidelines was a significant milestone in the development of a worldwide standard for the safety of cosmetics and personal care products. In particular, the standard focuses on technical, human, and administrative factors that can influence the quality of cosmetic products. Against this backdrop, the current study aimed to work towards a national consensus on cosmetics and personal care product safety by developing an instrument to prepare for cosmetic contract manufacturer auditing. Similar GMP questionnaires have been shown to be a useful aid in preparing for audits^14^, and numerous cosmetics manufacturers already employ such instruments. Nonetheless, thus far no study has investigated the use, contents, and validity of these GMP questionnaires.

The findings of the current study demonstrate that the developed audit preparation instrument has reasonably good content validity. Nearly all the quality items (47 of 52; 90%) were rated as essential by all the experts on the panel; the remaining 5 items did not meet the pre-defined criterion of at least 75% agreement. Based on these findings, 5 items were judged not relevant to the instrument and were excluded. While the Delphi method conventionally suggests a new Delphi questionnaire round at this point to conduct an additional assessment of agreement among the experts^19^, this study stipulated the criterion that if the agreement level was found to be below 75%, the particular item would be removed from the instrument. Hence, no further Delphi questionnaire rounds were performed.

The first draft audit in the brainstorming session received some criticism from the members of the scientific committee. Specifically, seven of the experts said there was too much detail in some of the questions, while others felt that the yes/no format provided insufficient information. Hence, these experts recommended using open-ended questions. Based on this feedback, text fields located at the end of each section was provided in the final audit instrument where the experts could clarify their responses in the Delphi round. However, in the early stages of the study, the case company decided that convenience in completing the audit preparation instrument was paramount, and thus yes/no questions were mostly used in its development.

A thorough knowledge base is necessary for the development of a GMP questionnaire^29^. However, the expert panel consulted in the Delphi round was relatively small (n = 50). Despite its small size, each panel member had experience in both GMP and the cosmetic industry, which gave them an in-depth perspective on the context of the research. Further, the inclusion of experts from the authorities and universities ensured the diversity of the panel. Finally, the Delphi questionnaire round received a very good response rate of 71.4%. In prior research, Delphi rounds involved expert panels ranging from 4 to 3000 individuals^17^, while several studies using the Delphi method involved relatively small expert panels^18,21^.

Building and Facilities section of the audit instrument ensures that the locations where products are manufactured and stored do not interfere with the quality of cosmetic products. The building and facilities section provides plant design and construction principles. It sets sanitary conditions under which manufacturers need to develop and store cosmetic products^30^**.** Through these principles, consumers can enjoy products that have undergone proper storage, adequate cleaning, and protection from toxic surfaces and environments. At the same time, the section has principles guiding the environment in which workers operate. Further, they emphasize ample water supply, proper sewage disposal, toilet facilities, hand-washing amenities, and rubbish clearance. Notably, the section ensures that consumers use cosmetic products manufactured under hygienic conditions.

The equipment section of audit instrument contains principles that guide the maintenance of utensils used in manufacturing, storing, and distributing cosmetic products. Precisely, the principles guide how manufacturers and distributors need to handle any type of equipment in relation to the contamination of cosmetic products. It emphasizes that cosmetic products need to stay clean to avoid contaminating products during manufacturing, storage, and distribution^30^**.** Therefore, consumers use products that are free from toxins and impurities originating from equipment used.

The personnel section of audit instrument involves every individual who handles cosmetic products at any level of production or distribution. This section stipulates that cosmetic manufacturers need to implement guidelines and programs that ensure disease control, cleanliness among all workers, proper education and training, and adequate supervision^30^**.** That way, consumers access products whose manufacture involves hygiene, expertise, and keen observation. Therefore, the personnel section of GMP is essential in ensuring those involved in the manufacture, storage, and distribution respect the health and safety of consumers.

Raw Materials section of audit instrument have numerous provisions. First, raw materials need to undergo inspection and be handled in a manner that ensures cleanliness^31^**.** The measure enhances a clean start during the manufacturing process. Second, raw materials need not contain microorganisms that cause disease in humans^31^**.** This guideline helps to protect consumers from using harmful cosmetic products. Third, materials with ingredients that are susceptible to contamination, such as aflatoxin, need to comply with the Drug Administration policies. This guideline protects consumers from using products with high levels of toxic chemicals. The final guideline is that raw materials need to stay in storage facilities that protect them from contamination^31^**.** The guideline ensures the use of clean raw materials for manufacturing. All the policies ensure that the raw materials become end-products that are safe for consumers’ bodies.

Production section has provisions that guide the conditions under which companies manufacture cosmetic products. The section emphasizes the appropriate time, temperature, pH, and acidification^32^**.** The section guidelines ensure that each product’s production process follows the recommended conditions. Consumers enjoy products whose production entailed the use of appropriate conditions.

Laboratory controls section involve testing the chemicals involved in the production of cosmetic products to check the levels of each chemical in the ingredients. With that in mind, laboratory control guidelines demand that components undergo investigation, sampling, retesting if needed, and thorough results analysis^33^**.** The guidelines ensure that consumers use products that are scientifically proven to be safe for human use.

Documentation section ensures traceability of all development, manufacturing, testing, and distribution activities^34^**.** The guidelines in this section ensure that whenever consumers need to ask about the manufacture of a product, the company will conveniently retrieve the records indicating its journey from development to sale.

The packaging and labeling section ensures that end-products are protected from contamination and mixing^31^**.** The main aim of the section is to protect the consumer from using cosmetic products that have toxins or are mixed up due to poor packaging and wrong labeling.

Complaints section demands that companies need to record and review each complaint^35^**.** The complaints will prompt the companies to recall or re-strategize the production of the involved cosmetic products. The section gives a voice to consumers since it ensures that manufacturers meet their quality demands.

Audit preparation instruments, such as the one developed here, have been shown to offer considerable support during the contract manufacturer auditing process. The current research will continue by investigating how well a completed audit preparation instrument reflects the reality during an audit. To achieve this, the answers provided by the company will be compared to the actual conditions observed while auditing. This comparison will offer valuable information on the applicability and practicality of the preparation instrument that can be integrated into future research. Furthermore, gathering feedback from contract manufacturers on the process of completing the instrument will also give crucial information on its usefulness.

The study results offer guidance for cosmetic companies aiming to incorporate cosmetics GMP when outsourcing their production to contract manufacturers, thereby ensuring consistency. Cosmetic companies operating outside the UAE may also find these results useful for implementing cosmetics GMP, although these findings primarily focus on the UAE context. However, elaboration and validation of an instrument such as described in this study is only a fraction of what it takes to obtaining a reliable and adequate instrument to be applied in its respective field—further studies are necessary to assess the psychometric properties of the instrument.

## Conclusion

The developed audit preparation instrument showed good content validity, and the expert panel participating in the Delphi questionnaire round only had a few minor suggestions for modifications. The content validation of the preparation instrument was done via the modified Delphi method, which proved to be suitable. However, there was some criticism of the instrument, and additional research is needed to ensure maximum applicability and practicality. This initial study involved systematically developing an audit preparation instrument, and it achieved good results; this research direction is to be continued in future studies with the aim of producing a system for validated quality management evaluation.

## Data Availability

The datasets generated during and/or analysed during the current study are available from the corresponding author on reasonable request.
